# Hexosylceramides and Glycerophosphatidylcholine GPC(36:1) Increase in Multi-Organ Dysfunction Syndrome Patients with Pediatric Intensive Care Unit Admission over 8-Day Hospitalization

**DOI:** 10.3390/jpm11050339

**Published:** 2021-04-24

**Authors:** Mara Leimanis-Laurens, Emily Wolfrum, Karen Ferguson, Jocelyn R. Grunwell, Dominic Sanfilippo, Jeremy W. Prokop, Todd A. Lydic, Surender Rajasekaran

**Affiliations:** 1 Pediatric Critical Care Unit, Helen DeVos Children’s Hospital, Grand Rapids, MI 49503, USA; karen.ferguson@spectrumhealth.org (K.F.); dominic.sanfilippo@helendevoschildrens.org (D.S.); surender.rajasekaran@spectrumhealth.org (S.R.); 2 Department of Pediatric and Human Development, College of Human Medicine, Michigan State University, Life Sciences Bldg., 1355 Bogue Street, East Lansing, MI 48824, USA; prokopje@msu.edu; 3 Bioinformatics & Biostatistics Core, Van Andel Institute, Grand Rapids, MI 49503, USA; Emily.Wolfrum@vai.org; 4 Pediatric Critical Care Medicine, Emory University & Children’s Healthcare of Atlanta, Atlanta, GA 30322, USA; jgrunwe@emory.edu; 5 Department of Pharmacology and Toxicology, Michigan State University, East Lansing, MI 48824, USA; 6 Collaborative Mass Spectrometry Core, Department of Physiology, Michigan State University, East Lansing, MI 48824, USA; lydictod@msu.edu; 7 Office of Research, Spectrum Health, Grand Rapids, MI 49503, USA

**Keywords:** lipidomics, pediatrics, critical illness, multi-organ dysfunction syndrome, glycerolipids, glycerophosphatidylcholine, sphingolipids, sphingomyelin

## Abstract

Glycero- and sphingo-lipids are important in plasma membrane structure, caloric storage and signaling. An un-targeted lipidomics approach for a cohort of critically ill pediatric intensive care unit (PICU) patients undergoing multi-organ dysfunction syndrome (MODS) was compared to sedation controls. After IRB approval, patients meeting the criteria for MODS were screened, consented (*n* = 24), and blood samples were collected from the PICU at HDVCH, Michigan; eight patients needed veno-arterial extracorporeal membrane oxygenation (VA ECMO). Sedation controls were presenting for routine sedation (*n* = 4). Plasma lipid profiles were determined by nano-electrospray (nESI) direct infusion high resolution/accurate mass spectrometry (MS) and tandem mass spectrometry (MS/MS). Biostatistics analysis was performed using R v 3.6.0. Sixty-one patient samples over three time points revealed a ceramide metabolite, hexosylceramide (Hex-Cer) was high across all time points (mean 1.63–3.19%; vs. controls 0.22%). Fourteen species statistically differentiated from sedation controls (*p*-value ≤ 0.05); sphingomyelin (SM) [SM(d18:1/23:0), SM(d18:1/22:0), SM(d18:1/23:1), SM(d18:1/21:0), SM(d18:1/24:0)]; and glycerophosphotidylcholine (GPC) [GPC(36:01), GPC(18:00), GPC(O:34:02), GPC(18:02), GPC(38:05), GPC(O:34:03), GPC(16:00), GPC(40:05), GPC(O:36:03)]. Hex-Cer has been shown to be involved in viral infection and may be at play during acute illness. GPC(36:01) was elevated in all MODS patients at all time points and is associated with inflammation and brain injury.

## 1. Introduction

The human plasma lipidome has been well described in the last decade [1] with the launch of the Lipid Maps Consortium (www.lipidmaps.org). From this work, it was found that over 600 unique molecular species exist, with a further estimate in the hundreds of thousands [2], half of which consists of glycerolipids, glycerophospholipids and sphingolipids. All of these lipid classes are structurally constituted of a fatty acid with long hydrocarbon chains, and a glycerol group, with sphingolipids as the only exception lacking a glycerol group (from Quehenberger and Dennis [1]). Fatty acids are the building blocks of all lipids, and when in free form, are highly metabolically active, and famously linked to enzymatically regulated eicosanoid synthesis [3,4]. 

### 1.1. Multi-Organ Dysfunction Syndrome (MODS) and Role of Lipids in Critical Illness

Multi-organ dysfunction syndrome (MODS) has been previously described as a major source of pediatric intensive care unit (PICU) admissions [5], and fatalities [6]. MODS depends largely on the critical illness diagnosis, the etiology of which may include sepsis, chronic diseases, and patients’ age (neonates at highest risk). Both proinflammatory and anti-inflammatory mediators play a role, for which the lungs are often the first organ affected (heart, brain, kidney, and liver as additional organs affected). Clinical presentations of pediatric MODS conditions include thrombocytopenia-associated multiple organ failure, hemophagocytic lymphohistiocytosis, Kawasaki syndrome, and hematologic malignancies. Management includes ventilatory and hemodynamic support, prevention of renal damage, and/or administration of immunosuppressive agents [7]. A portion of the patients with MODS are due to infections, as most of our current understanding of the pathophysiology has been taken from work done in pediatric sepsis [8,9,10,11]. A smaller cohort of patients requires aggressive life support measures such as extracorporeal membrane oxygenation (ECMO), with 45% reported mortality [12].

More recently, in the nascent field of lipidomics, effects of infection from viruses have been described in plasma, namely for COVID-19 [13] and Ebola [14], as the specific assault on the patient appears to harbor different metabolic consequences for its victims. Perhaps the most well-characterized and described of all the lipids are plasma glycerolipids, namely triacyglycerides (TAGs), which are famously involved in many metabolic syndromes [15,16], along with diacylglycerols (DAGs) [17]. TAG’s have been found to be elevated in children with systemic inflammatory response syndrome and sepsis [15] and in adults with acute respiratory distress syndrome [16]. 

Phosphatidylcholine (PC) (lecithin) is a membrane phospholipid, which is required for some membrane-associated enzymes, aids in cholesterol transport through the cell [18], and is highly abundant in plasma. A recent report characterized PC abundance in a healthy French population of volunteers (N = 800) [19], which found 76/186 plasma metabolites to be PC. Stated differently, as described by Quehenberger et al., PC constitutes 76% of total glycerophospholipids [4] and with a glycerol sidechain, glycerophosphatidylcholine (GPC) is formed. However, very little is reported on GPC’s exact biological function and association to disease states.

Sphingolipids exhibit both structural and signaling function (ceramides), and unlike other lipids with a glycerol backbone, consists of a sphingoid backbone. Ceramides are intermediates in sphingolipid metabolism and regulate cellular pathways including apoptosis, cell senescence, and cell cycle and differentiation [20], and are involved in the cell’s response to stress [21] (and further produce sphingomyelins). In general, sphingolipids have been characterized in infection, autoimmunity and neurodegenerative disorders. Sphingolipids and the sphingolipidome have been recently characterized in the involvement in hemophagocytic lymphohistiocytosis [22], linked to Alzheimer’s [23], and inflammation [24]. Given the existing literature, we anticipate seeing differences in these lipid classes in our patients. 

### 1.2. Lipidomics as Biomarkers in Critical Illness

Little is known on the dyslipemia during the height of critical illness. From a previous report [25], we found that phospholipids were highly down-regulated in the acute phase of illness, as patients were being admitted to the PICU, and partially recovered with the introduction of feeds by the eight-day mark. Based on these initial findings, we have expanded our analysis to further evaluate the potential effects of critical illness on a broader range of lipids, including glycerolipids, glycerophosphatidylcholine, sphingolipids and sphingomyelins over an eight-day PICU course. 

## 2. Results

### 2.1. Study Description and Demographics 

Basic demographics and dietary intake were previously reported [25,26]. In brief, we had a total of 61 patient samples over three time points for 28 patients. All patients were being mechanically ventilated at the time of the baseline sample collection and receiving inotropes. Additional organs affected were the kidneys (*n* = 15; 54%), including 7 MODS and all 8 ECMO patients; the liver (*n* = 8; 28.6%; 4 MODS; 4 ECMO), and the brain (*n* = 6; 21.4%; 4 MODS; 2 ECMO). The severity of illness scores (Pediatric Logistic Organ Dysfunction-2 score (PELOD)) were not significantly different among groups across time points (Global F-stat, *p* = 0.66). 

### 2.2. Percent Total Lipids over Time and Treatment Course 

#### 2.2.1. Sphingolipids and Glycerolipids

Lipid percentages for glycerolipids (including TAG’s) and sphingolipids are listed in Table 1. Phospholipids constitute the vast majority of plasma lipids and were described in a previous report [25]. The second major lipid group in terms of total percentage includes the sphingolipids with mean value of 8% for MODS at baseline, which is much lower than 13% for sedation controls (*t*-test, *p* = 0.0056). At eight days, the mean value is 15% for the MODS group, indicating a dramatic doubling over the eight-day course. ECMO patients demonstrated an intermediate profile. Glycerolipid values in all patients were higher than sedation controls by day eight, and are typically the major lipid in plasma. Sphingolipids were low at baseline and increase over time; the glycerolipids are already higher at baseline and increased at eight days. 

#### 2.2.2. Sub-Groups of Sphingolipids

From these initial observations of total plasma sphingolipids, we were able to quantify structurally varied groups, namely dihydrosphingomyelin (dhSM), sphingomyelin (SM), ceramides (Cer), 2-hydroxy Ceramides (2-hydroxy Cer), Hexosylceramides (Hex-Cer), lactosylceramides (Lac-Cer) (Table 2). The overwhelming majority of the sphingolipids were SMs with median ranges in sick patients from 95% compared to 99.8% in our sedation control patients. All of the MODS patients had significantly fewer SMs as compared to sedation controls when analyzed using a *t*-test over the three time points with p-values ranging from <0.0023–<0.0001. This is likely what contributed to the low levels of sphingosine. Both dhSM and 2-hydroxy Cer had below detectable limits in the sedation controls, making the comparatives impossible. 

DhSM, which has been shown to contribute to membrane rigidity inhibiting viral-cell membrane fusion [27], was detected in ECMO patients only at all three time points with mean ranges of 0.75–1.39%, the maximum level was achieved at eight days. 2-hydroxy Cer which is a precursor to ceramide and been associated with pro-apoptotic activity [28] was detected at 72 h and eight days with mean ranges from 0.17% to 0.48% detected per group. One metabolite of ceramide Hex-Cer was higher in the MODS patients across all time points with mean percent differences ranging from 1.63–3.07% vs. 0.22% (*t*-test, *p*-values: 0.003–<0.0001), values continued to increase at eight days, at which time ECMO patients also demonstrate a statistical difference (*t*-test, *p*-value: <0.001). Lac-Cer has been recently shown to have a protective effect for type II diabetes [29], and seems to follow a similar patterning to Hex-Cer with elevated mean percent values at 72 h for MODS patients 0.37 (*t*-test, *p* = 0.0011), and ECMO patients at eight days (*t*-test, *p* = 0.0045). 

#### 2.2.3. Lipids Species for Sphingolipids and Glycerolipids at Baseline and over Time

**Figure 1:**
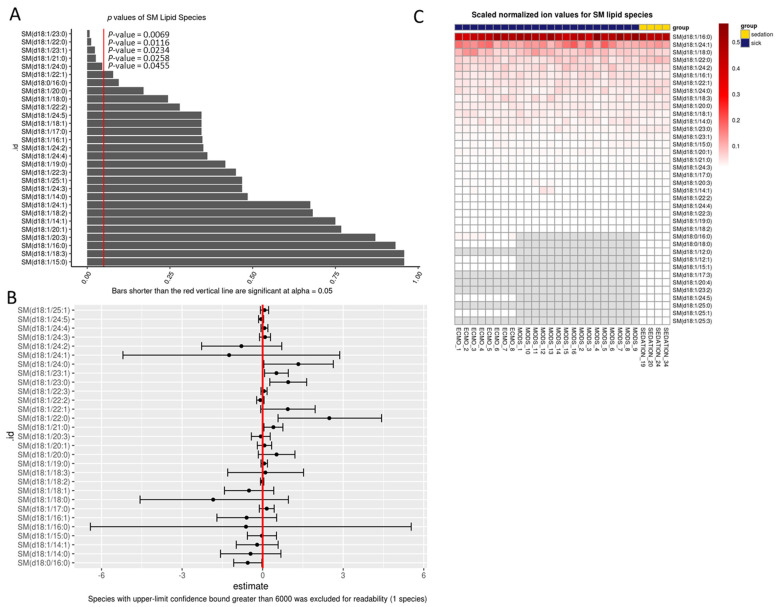
Percent total sphingomyelin lipid species at baseline. (**A**) Bar chart of *p*-values from regression output from log-transformed data; red line indicates 0.05. Any *p*-value that crosses 0.05 is not statistically significant. (**B**) Points in B represent the mean log(fold-change) in odds between sick and sedation groups. The error bars in B, represent the upper and lower bounds of the false-coverage for the estimate. 95% false coverage intervals of the mean log (fold-change) in odds; red line indicates 0. Any confidence interval that crosses 0 is not statistically significant. (**C**) Scaled values of total lipids based on absolute normalized ion abundances per ml of plasma; SM: sphingomyelin.

We were interested in reviewing lipids at the species level. This allowed us to determine the length of the backbone carbon chain and the number of double bonds (level of lipid saturation). Only the baseline samples were analyzed, given that patients were nil per os (NPO), and nutrition intervention was minimized. A total of five SM species were found to be statistically significantly different as compared to sedation controls when analyzed using a generalized linear model that was adjusted for age and sex, SM(d18:1/23:0; *p* = 0.007), SM(d18:1/22:0; *p* = 0.012), SM(d18:1/23:1; *p* = 0.023), SM(d18:1/21:0; *p* = 0.03), SM(d18:1/24:0; *p* = 0.05) (Figure 1A,B). All except one SM(d18:1/23:1) was with saturated carbon-chains, and no double bonds. The heatmap shows generally less variability in sick patients (Figure 1C). 

**Figure 2:**
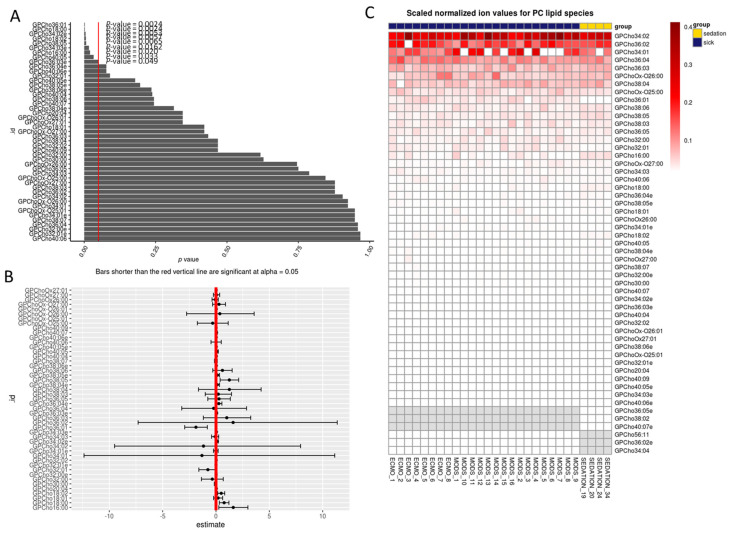
Percent total glycerophosphotidylcholine lipid species at baseline. (**A**) Bar chart of *p*-values from regression output from log-transformed data; red line indicates 0.05. Any *p*-value that crosses 0.05 is not statistically significant. (**B**) Points in B represent the mean log (fold-change) in odds between sick and sedation groups. The error bars in B, represent the upper and lower bounds of the false-coverage for the estimate. 95% false coverage intervals of the mean log (fold-change) in odds; red line indicates 0. Any confidence interval that crosses 0 is not statistically significant. (**C**) Scaled values of total lipids based on absolute normalized ion abundances per ml of plasma; GPC: glycerophosphotidlycholine.

A total of nine glycerophosphotidylcholine (GPC) species were found to be significantly different in critically ill children compared to sedation controls when analyzed with a generalized linear model that was adjusted for age and sex, GPC(36:01; *p* = 0.002), GPC(18:00; *p* = 0.002), GPC(O:34:02; *p* = 0.005), GPC(18:02; *p* = 0.006), GPC(38:05; *p* = 0.007), GPC(O:34:03; *p* = 0.016), GPC(16:00; *p* = 0.020), GPC(40:05; *p* = 0.033), GPC(O:36:03; *p* = 0.05) (Figure 2A,B). With the exception of GPC(36:01), all of the other eight GPC species were lower than sedation controls. Choline is a marker of cellular integrity, membrane damage and turnover. The heatmap reveals a complete absence of certain species such as GPC(34:01), which implies a vast diversity of lipid species (Figure 2C). 

Additional generalized linear model analysis was run on Cholesterol (Chol) and cholesteryl esters, and TAGs, which did not reveal any statistically significant changes between the sick and sedation controls at the lipid species level, and were not further reviewed (Supplementary Figures S1 and S2). Diglycerides did reveal one single species (DG36:01) in the sedation control group (Supplemental Figure S3), a patterning which was lower for both MODS and ECMO patients.

**Figure 3:**
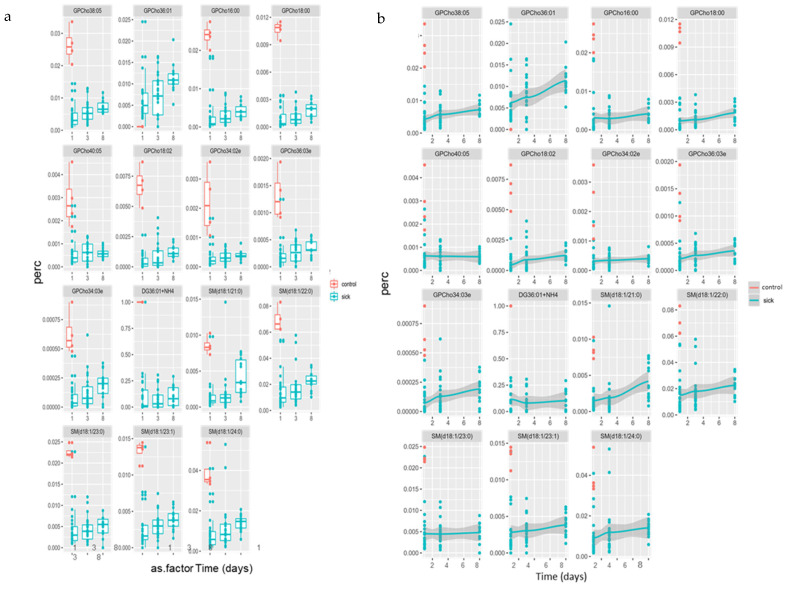
(**a**,**b**) Time course for percent GPC and SM species lipids over 8 days; (**a**) boxplots; (**b**) geometric mean.

Nutrition intervention on remodeling of the lipidome was determined by 72 h for all patients. Visualization of changes in key lipid species from Figure 1 and Figure 2 over time, are summarized in Figure 3a (as boxplots) and Figure 3b (as geometric mean). Generally, all lipid species were increasing over time. This may indicate a shift from the acute phase of illness into recovery, as in the case of SM(d18:21:0); however, the majority remain lower as compared to sedation controls. Consequently, one GPC species, GPC (36:01), was found to be higher than the sedation controls in Figure 2, also increased over time. 

### 2.3. Correlations Analysis

In an attempt to better attribute the statistical differences in sphingolipid levels with physiological conditions, we ran a correlation analysis over the three time points as compared to percent total calories, percent total protein, brain injury (yes/no) and infection (bacterial/viral) (Table 3a,b; Supplementary Tables S1 and S2). ECMO patients at day eight demonstrated a negative correlation to brain injury for total dhSM (−0.730), total SM (−0.730), total Hex-Cer (−0.730), and total Lac-Cer (−0.730) (Table 3a). Similarly, ECMO patients at day eight positively correlated with total protein intake for total dhSM (0.733), and total Lac-Cer (0.867), moderately with total SM (0.600), and total Hex-Cer (0.600); moderately positively correlated at day 1 for total Cer (0.600), and at day three for total 2-hydroxy Cer (0.683) (Table 3b). The sample sizes at three time points for ECMO patients were small (*n* = 6), therefore the interpretation of this result must be tempered. However, nutritional intake may be the most strongly correlated modifier we explored. The same partial Pearson correlation to examine the relationship between GPCho36:01 and brain abnormality (0/1) was used, and adjusted for age, group status (ECMO/mods/sedation), and day (1, 3, 8). The estimated partial correlation is 0.28 between the two variables (brain_abnormal, GPCho36:01). Unless the r2 came back as 0, we cannot say that there is no correlation. There is a correlation, it is small and not significant at our *p* < 0.05 cut-off, we cannot speculate about the clinically relevance.

## 3. Discussion

Sphingolipids are fundamental to the structural components of cell membranes and are involved in the regulation of biological processes including cell growth, differentiation and apoptosis. Sphingolipids were found to be lower at baseline in our patients and increased over time. In our patient population, levels of plasma Hex-Cer, a metabolite of ceramide, were found to be higher than that of sedation controls over the course of the eight days. Lipid rafts formed through the interaction of cholesterol and glycosphingolipid, creating platforms to support signal transduction, pathogen infection [30], and the viral life cycle [31]. Hex-Cer has been shown to play a role in chronic hepatitis C infections [32] and viral replication. We found that 14/24 of our study participants had infections. We believe that our lipidomics results were linked to an infection; however, the correlation analysis did not reveal a strong correlation to viral or bacterial infection for the sub-groups of SMs (Supplementary Table S2). 

The common denominator in our patient cohort is inflammation and trauma, as other patients included admission diagnoses of: burn victim, carbon monoxide poisoning, cardiac conditions (*n* = 2), acute kidney disease and others as a subset of patients who required ECMO, which is a life-saving intervention [33]. However, these drivers of progression from MODS to ECMO are poorly understood. Generally, work in the sepsis population has been better explored by Wong et al. [9,10]. 

The detailed etiology of the patient population was outlined in previous publications from this cohort, namely the transcriptome (Prokop et al., 2020 [34], Shankar et al., 2020 [26]), which revealed that decreased neutrophils were correlated to an increased risk of progression from MODS to ECMO. In two additional reports on this patient cohort, namely on the plasma metabolites-non-polar and hydrophilic (Leimanis-Laurens, et al., 2021 [35]), and polar-hydrophobic-lipidome (phospholipids) were further reviewed (Leimanis-Laurens, et al., 2021 [25]). This latter study correlated phospholipids to nutritional intake (percent calories; percent protein) and suggested a role for neutrophils, which are reduced in number during times of immunosuppression (as bone marrow activity is suppressed). Frasch et al. outlined in detail the relationship between lysophosphatidyserine (lysoPS) [36], which was very low in our patient cohort [25], and neutrophil-mediated efferocytosis (“eat me”-cradle-to-grave signal for macrophages during acute inflammation [37]). All of the SM species were low in comparison to controls, which corresponds to the overall patient profiles as shown by the low percentage of SP and SMs found in the critically ill cohort.

There was a large amount of variability in the glycerolipids, with time points higher than controls. Glycerolipids (mono- di- tri- glycerides) are largely exogenous in nature and obtained through dietary consumption. Most believe that TAG’s are purely for energy storage, therefore this increase in glyerolipid levels may not be surprising given the catabolic state of critically ill patients. Sepsis also causes hyper-triglyceridemia [38], which may have contributed to the spike in TAG levels. By contrast, lipotoxicity may result from an increase in TAGs or the breakdown product of TAGs, non-esterified fatty acids (NEFA’s) [25], and TAG/TG metabolites (ceramides, diacylglycerols) [39,40]. 

One result was striking. GPC(36:1), which has been associated with human glioma [41], and was abundant in all sick patients. One of the building blocks of GPC(36:1) is oleic acid (18:1), which is a pre-cursor to linoleic acid that feeds into the generation of arachidonic acid. Levels of oleic acid and arachidonic acid have been shown to be inversely correlated [42,43]. Some mammals lack the ability to convert linoleic acid to arachidonic acid, therefore obtaining polyunsaturated omega-6 fatty acid from dietary animal sources (meat, eggs) is essential. Choline levels increase in brain tumors and inflammation. This marker may imply that our critically ill patients are undergoing some type of brain injury, beyond that which is visible by routine neurological monitoring. 

In light of recent findings with COVID, the recovery from metabolic alterations can take time, as patients discharged from the hospital irrespective of severe or mild symptoms, had not yet re-normalized their metabolite and lipid levels [13]. Our data show how critical illness affects lipid metabolism, with subsequent recovery possible by dietary intervention for glycerolipids and sphingolipids. 

Our study has several limitations. Large variability in the lipidomic signature of a limited number of samples is present, limiting our statistical analysis; therefore, we can only comment on the overall increases and decreases in the data. This was an observational study and thus we are unable to infer that changes in lipids over time are the result of clinical interventions, such as the introduction of enteral feeds. Due to the many variables measured and the limited sample size, there is a possibility of Type 1 errors, and false-positive results [44], which are characteristic of high dimensional data analyses. The metabolome-wide significance level (MWSL) (*p* > 2 × 10^−5^–4 × 10^−6^) should be adopted in future studies [45]. Additional confounding factors, such as pre-admission diet, medication use, medical status, and inherent metabolic and genetic differences likely exist but were not measured in this study. 

## 4. Materials and Methods

### 4.1. Design, Site, Sample and Data Collection

Critically ill patients admitted to the PICU in Western Michigan, and referred by the attending physician, were screened, consented, and had blood samples collected up to three independent time points (baseline, 72 h, and 9 days), according to IRB approval (2016-062-SH/HDVCH), as previously described [25,26,34,35]. The authors obtained parental informed consent prior to recruitment into the study. If patients were discharged before the later time points, samples were not collected. Helen DeVos Children’s Hospital PICU sees 1500 admissions per year (over 6000 patient-days), has seventeen board-certified intensivists covering a 24-bed unit with flex capability to care for up to 36 critically ill children, including a cardiothoracic surgery program and a level 1 trauma center. Patients with MODS fit the following inclusion criteria: <18 years of age; on vasopressors with a central line and requiring respiratory support. Sedation control patients were presenting for routine sedation with benign characteristics; samples were collected one time. Patients were excluded if they had a known autoimmune disease, had a limitation of care in their advance directives, had undergone cardiopulmonary bypass prior to onset of MODS, received plasmapheresis prior to ECMO initiation, and were patients of the neonatal intensive care unit. Blood samples were drawn in EDTA-filled anticoagulant, centrifuged and stored at −80 °C. Basic demographic variables were pulled from the local electronic medical record (EMR). Dietary history was extracted from the dietician’s EMR, as previously reported [25]. Data were collected and managed using REDCap [46]. The severity of illness scores were retrieved through the Virtual Pediatric Intensive Care Unit Performance Systems (VPS, LLC, Los Angeles, CA, USA). Nutritional intake was previously described [25].

### 4.2. Blood Plasma Lipidomics Method

Blood samples collected in EDTA-treated tubes were immediately placed on ice and then spun at 4 °C (once for 15 min at 1500 rpm; a second spin for 10 min at 10,000 rpm), plasma was harvested and frozen to −20 °C, and −80 °C for long-term storage (as previously described [25]). Lipidome profiles were determined from five microliters of plasma thawed on ice, while the remainder of the plasma sample was reserved for other studies. Each 5-microliter aliquot was diluted in 95 microliters of HPLC-grade water and subjected to lipid extraction in 2 mL glass tubes with PTFE-faced caps, using an extraction mixture of with acetone, methanol, and acetonitrile (1:1:1, *v*:*v*:*v*) according to [47]. Lipid extracts were dried under nitrogen and reconstituted in isopropanol:methanol:chloroform (4:2:1, *v*:*v*:*v*) by gentle vortexing for 1 min. Di-myristoyl phosphatidylcholine was spiked into each sample during extraction as an internal standard, such that the final concentration was 0.5 picomole/microliter in the reconstituted lipid extracts. Immediately prior to mass spectrometric analysis, aliquots of each plasma lipid extract were loaded into an Eppendorf 96-well plate and evaporated under nitrogen. The lipids were then resuspended in a solvent of 20 mM ammonium formate in isopropanol:methanol:chloroform (4:2:1, *v*:*v*:*v*) and the 96-well plate was sealed with a sealing mat (Analytical Sales and Services, Flanders, Belgium). The 96-well plate was then loaded into an Advion Nanomate Triversa (Advion Biosciences, Ithaca, NY, USA) that served as the nano-electrospray ionization source and high-throughput autosampler. The autosampler temperature was held at 12 °C during the analysis. The Nanomate spray voltage was held at 1.4 kV and a gas pressure of 0.3 psi. Under these conditions, the Nanomate operates at an nESI flow rate of approximately 500 nanoliters per minue. Five microliters of each lipid extract was directly infused into an LTQ-Orbitrap Velos mass spectrometer (Thermo Scientific, Waltham, MA, USA) with the FT analyzer operating at 100,000 resolving power (defined at *m*/*z* 400) and a scan rate of 1 Hz. Full MS scans were collected for one minute each in positive and negative ionization modes. The inlet of the mass spectrometer was held at 100 °C, the S-lens was set to 50 percent, and the trap accumulation time was 300 milliseconds. Under these conditions, in-source fragmentation is minimal under nESI conditions. To verify lipid identities of abundant lipids, ion mapping MS/MS was performed on pooled lipid extracts using higher-energy collisional dissociation at a normalized collision energy of 60 and 100,000 resolving power, at a step size of 1.0 mass units between *m*/*z* 200 and 1000, and a trap accumulation time of 1000 milliseconds. Prior to MS data collection, mass calibration was performed on the FT analyzer according to the vendor’s instructions using an automated calibration routine. Following initial data collection, each mass spectrum was additionally subjected to offline mass recalibration using the Xcalibur software (ThermoFisher Scientific, Waltham, MA, USA; version 2.2) Recal Offline tool in order to further refine mass accuracy and eliminate drift in instrument calibration over the duration of the analytical run. The peak findings, correction of ^13^C isotope effects, and quantification for global lipidomics and targeted lipid mediators was performed with Lipid Mass Spectrum Analysis (LIMSA) version 1.0 software, Helsinki, Finland [48] as previously described [49]. The software vendor’s “linear fit” algorithm was used for isotope correction, and a mass search window of 0.003 *m*/*z* was utilized to match MS1 peaks to known endogenous lipids and the spiked synthetic internal standard.

All calculated peak areas of found peaks were normalized to that of the internal standard. Due to the untargeted nature of the analysis, no attempts were made to quantitatively correct for differences in ionization efficiencies across lipid species owing to length and degrees of unsaturation of the lipid acyl chains or the polarities of lipid headgroups. All quantitated found lipid peak data from separate positive and negative ion analyses were subsequently combined in Microsoft Excel software for the purpose of downstream data analysis and statistical evaluation. Lipidome analysis provided untargeted assessment across all classes of glycerolipids (GC) (including mono-, di- and triglycerides), phospholipids (PS), glycerophosphatidylcholines (GPC’s) (species; GPCho34:02; GPCho36:02; GPCho34:01; GPCho36:04; GPCho36:03; GPChoOx-O26:00; GPCho38:04; GPChoOx-O25:00; GPCho36:01; GPCho38:06; GPCho38:05; GPCho38:03; GPCho36:05; GPCho32:00; GPCho32:01; GPCho16:00; GPChoOx-O27:00; GPCho34:03; GPCho40:06; GPCho18:00; GPCho36:04e; GPCho38:05e; GPCho18:01; GPChoOx26:00; GPCho34:01e; GPCho18:02; GPCho34:01e; GPCho38:04e; GPChoOx27:00; GPCho38:07; GPCho32:00e; GPCho30:00; GPCho40:07; GPCho34:02e; GPCho36:03e; GPCho40:04; GPCho32:02; GPChoOx-O26:01; GPChoOx27:01; GPCho38:06e; GPChoOx-O25:01; GPCho32:01e; GPCho20:04; GPCho40:09; GPCho40:05e; GPCho34:03e; GPCho40:05e; GPCho36:05e; GPCho38:02; GPCho40:07e; GPCho56:11; GPCho36:02e; GPCho34:04), lyso-phospholipids (lysoPL), sphingolipids (SP), sterols, non-esterified fatty acids (NEFA’s) and fatty acids (FAs). Additional species analysis was completed on phospholipids (PL), triacylglycerides (TGs), diacylglycerides (DGs), cholesterol (chol), and sphingomyelins (SMs) [species; SM(d18:1/16:0); SM(d18:1/24:1); SM(d18:1/18:0); SM(d18:1/22:0); SM(d18:1/24:2); SM(d18:1/16:1); SM(d18:1/22:1); SM(d18:1/24:0); SM(d18:1/18:3); SM(d18:1/20:0); SM(d18:1/18:1); SM(d18:1/14:0); SM(d18:1/23:0); SM(d18:1/23:1); SM(d18:1/15:0); SM(d18:1/20:1); SM(d18:1/21:0); SM(d18:1/24:3); SM(d18:1/17:0); SM(d18:1/20:3); SM(d18:1/14:1); SM(d18:1/22:2); SM(d18:1/24:4); SM(d18:1/22:3); SM(d18:1/19:0); SM(d18:1/18:2); SM(d18:0/16:0); SM(d18:0/18:0); SM(d18:1/12:0); SM(d18:1/12:1); SM(d18:1/15:1); SM(d18:1/17:3); SM(d18:1/20:4); SM(d18:1/23:2); SM(d18:1/24:5); SM(d18:1/25:0); SM(d18:1/25:1); SM(d18:1/25:3)]. Large blood volumes from this patient population was challenging to obtain; therefore, plasma volumes were small (~0.050–0.075 mL total). As only a small fraction of each sample was available for lipidomic analysis, primarily and higher abundance lipids were targeted [50].

### 4.3. Analysis

The decision was made a priori to compare the sedation control population (*n* = 4) with all of the critically ill children (*n* = 24), unless stated otherwise. Some statistical analyses were performed using R (v 3.6.0, Vienna, Austria) [51]. Where appropriate, percent data were transformed before being analyzed with a beta regression from the R package betareg [52], and MedCalc (MedCalc Software Ltd., Ostend, Belgium). Total normalized ion values were log-transformed and then analyzed using generalized linear regression models (glm) [53]. All regression models were adjusted for age and sex. Contrasts between treatment groups (sick vs. sedation) were conducted using R package emmeans [54]. P-values from regression analyses have been corrected for multiple testing via the FDR method. We focused on glycerol- glycerophospho- and sphingo-lipids. Additionally, independent *t*-tests, Welch’s *t*-tests, and F-tests of equal variances were run on these data and included multiple testing adjustments. Correlation analysis was performed where indicated using Kendall Tau, whereby percent calories and protein intake were calculated from resting energy expenditure [55], and daily required intake [56] (≤33%; 34–66%; and ≥67%), binary values (yes/no) for brain injury and infection (bacterial/viral).

## 5. Conclusions

We note a low abundance of sphingolipids and sphingomyelins for this cohort of critically ill pediatric patients. Trace amounts of markers that are pro-apoptotic and associated with membrane homeostasis were also found. A ceramide metabolite previously associated with infection Hex-Cer, was elevated, in concert with Lac-Cer an intermediary in glycosphingolipid metabolism may have a protective effect against infection. In total, our observations display a complex interplay of lipids, with a lipid species of GPC(36:1) highlighted as a potential biomarker candidate of critical illness. The pathways for biosynthesis of these lipids are well known, future work will involve exploration of these pathways. 

## Figures and Tables

**Table 1:** Percent total of major lipid classes of MODS/ECMO Study 2016–2018 (*N* = 28).

Lipid Class	Sphingolipids				Glycerolipids			
	Mean	SD	Median	*p*-Value	Mean	SD	Median	*p*-Value
Sedation Controls (*n* = 4)	13.03	1.24	13.21		3.43	3.34	2.57	
MODS BL (*n* = 16)	7.86	3.18	7.40	*p* = 0.0056	12.25	13.35	5.39	*p* = 0.0296 *
ECMO BL (*n* = 8)	12.32	2.59	11.91	*p* = 0.6217	10.10	8.63	8.37	*p* = 0.1746
MODS 72 h (*n* =15)	9.06	3.82	9.88	*p* = 0.0601	9.58	7.16	6.75	*p* = 0.1182
ECMO 72 h (*n* = 7)	11.50	3.22	10.80	*p* = 0.3952	8.51	9.20	4.19	*p* = 0.3234
MODS 8d (*n* = 8)	14.85	3.71	15.20	*p* = 0.3713	10.01	6.10	9.59	*p* = 0.0759
ECMO 8d (*n* = 6)	11.36	2.33	11.16	*p* = 0.2305	11.67	5.48	11.80	*p* = 0.0285

Notes: All tests are using sedation as comparative group; *t*-test performed (assuming equal variances); * F-test for equal variances was *p* ≤ 0.05 Welch test (assuming unequal variances) was used. Using Bonferroni correction for multiple comparisons (significance *p* ≤ 0.0083); BL: baseline; 72 h: 72 h; 8d: 8 days.

**Table 2:** Percent total sphingolipids of MODS/ECMO Study 2016–2018 (*N* = 28).

Lipid Class	dhSM				SM				Cer				2-hydroxy Cer				Hex-Cer				Lac-Cer			
	Mean	SD	Mdn	*p*-Value	Mean	SD	Mdn	*p*-Value	Mean	SD	Mdn	*p*-Value	Mean	SD	Mdn	*p*-Value	Mean	SD	Mdn	*p*-Value	Mean	SD	Mdn	*p*-Value
Sedation (*n* = 4)	0.00	0.00	0.00		99.71	0.25	99.79		2.54	1.52	2.32		0.00	0.00	0.00		0.22	0.25	0.15		0.04	0.03	0.05	
MODS BL (*n* = 16)	0.00	0.00	0.00	N/A	95.22	3.98	96.33	*p* = 0.0004 *	3.04	3.51	1.79	*p* = 0.79	0.00	0.00	0.00	N/A	1.63	1.57	1.19	*p* = 0.0033 *	0.11	0.10	0.09	*p* = 0.0210
ECMO BL (*n* = 8)	0.75	0.62	1.15	N/A	96.66	2.64	97.39	*p* = 0.0143 *	1.98	1.38	1.88	*p* = 0.53	0.17	0.20	0.09	N/A	2.41	2.35	1.27	*p* = 0.0347 *	0.15	0.12	0.09	*p* = 0.0367 *
MODS 72 h (*n* = 15)	0.00	0.00	0.00	N/A	95.17	1.79	95.50	*p* < 0.0001 *	1.49	1.04	1.21	*p* = 0.12	0.25	0.43	0.06	N/A	2.73	1.59	2.46	*p* < 0.0001 *	0.37	0.31	0.31	*p* = 0.0011 *
ECMO 72 h (*n* = 7)	1.05	1.05	1.15	N/A	95.67	2.12	96.77	*p* = 0.0025 *	1.02	0.51	0.85	*p* = 0.15	0.41	0.32	0.56	N/A	3.19	2.65	1.97	*p* = 0.0260 *	0.06	0.05	0.06	*p* = 0.3609
MODS 8d (*n* = 8)	0.00	0.00	0.00	N/A	96.03	1.22	95.91	*p* < 0.0001 *	0.37	0.25	0.35	*p* = 0.07	0.32	0.27	0.37	N/A	3.07	1.13	3.18	*p* = 0.0001 *	0.21	0.10	0.21	*p* = 0.0090
ECMO 8d (*n* = 6)	1.39	1.09	1.49	N/A	95.12	1.95	94.98	*p* = 0.0023 *	0.66	0.52	0.51	*p* = 0.10	0.48	0.77	0.12	N/A	3.05	1.07	3.04	*p* = 0.0008 *	0.41	0.18	0.42	*p* = 0.0045

Notes: All tests are using sedation as comparative group; * Welch-test (assuming unequal variances); BL: baseline; 72 h: 72 h; 8d: 8 days; 2-hydroxy Cer: 2-hydroxy ceramide; Cer: ceramide; dhSM: dihydrosphingomyelin; SD: standard deviation; Hex-Cer: hexosylceramides; Lac-Cer: lactosylceramides; Mdn: Median; SM: Sphingomyelin.

**Table 3:** (**a**) Correlations between lipid values and brain injury (yes/no) in MODS patients over time, (**b**) Kendall correlations between lipid values and percent of total protein.

(**a**)							
**Group**	**Time**	**Total dhSM**	**Total SM**	**Total Cer**	**Total hydroxy Cer**	**Total Hex-Cer**	**Total Lac-Cer**
MODS	Day 1	NA	−0.053	−0.053	NA	−0.172	−0.238
MODS	Day 3	NA	−0.059	0.029	−0.217	0.029	0.074
MODS	Day 8	NA	0.071	−0.357	−0.218	0.214	0.214
ECMO	Day 1	−0.577	−0.655	−0.218	0.000	−0.546	−0.655
ECMO	Day 3	−0.149	−0.552	0.138	−0.414	−0.276	−0.354
ECMO	Day 8	−0.730	−0.730	0.183	−0.365	−0.730	−0.730
(**b**)							
**Group**	**Time**	**Total dhSM**	**Total SM**	**Total Cer**	**Total 2-hydroxy Cer**	**Total Hex-Cer**	**Total Lac-Cer**
ECMO	1.000	0.499	0.262	0.577	0.000	0.157	−0.052
ECMO	3.000	0.264	0.390	0.098	0.683	0.390	−0.250
ECMO	8.000	0.733	0.600	−0.067	−0.067	0.600	0.867
MODS	1.000	NA	NA	NA	NA	NA	NA
MODS	3.000	NA	NA	NA	NA	NA	NA
MODS	8.000	NA	−0.429	−0.071	−0.182	−0.357	−0.214

Notes: 2-hydroxy Cer: 2-hydroxy ceramide; Cer: ceramide; dhSM: dihydrosphingomyelin; Hex-Cer: hexosylceramides; Lac-Cer: lactosylceramides; SM: Sphingomyelin. Table 3a was not adjusted for sex and age.

## Data Availability

Data available upon request.

## Supplementary Materials

The following are available online at https://www.mdpi.com/article/10.3390/jpm11050339/s1, Table S1: Kendall Correlations between Lipid Value and Percent of Total Calories; Table S2: Correlations between lipid values and viral infection (yes/no) in patients over time; not adjusted for age or sex; Figure S1: Percent total cholesterol lipid species; Figure S2: Percent total triacylglycerol lipid species; Figure S3: Percent total diacylglycerol lipid species.

## Abbreviations

Cer: ceramide; chol: cholesterol; DAG’s: diacylglycerides; dhSM: dihydrosphingomyelin; ECMO; extracorporeal membrane oxygenation; EMR: electronic medical record; FA’s: fatty acids; GC: glycerolipids; GP: glycerophospholipids; GPC: glycerophosphatidylcholine; Hex-Cer: hexosylceramides; HLH: hemophagocytic lymphohistiocytosis; 2-hydroxy Cer: 2-hydroxy ceramide; Lac-Cer: lactosylceramides; MODS: multi-organ dysfunction syndrome; NADPH: nicotinamide adenine dinucleotide phosphate; NEFA’s: non-esterified fatty acids; NPO: nil per os; PICU: Pediatric Intensive Care Unit; PICU LOS: PICU length of stay; PELOD; Pediatric Logistic Organ Dysfunction-2 score; SD: standard deviation; SM: sphingomyelins; SP: sphingolipids; TAG’s: triacyl glycerides.
