# Supplementary material for: Hexosylceramides and Glycerophosphatidylcholine GPC(36:1) Increase in Multi-Organ Dysfunction Syndrome Patients with Pediatric Intensive Care Unit Admission over 8-Day Hospitalization
Source: J Pers Med. 2021 Apr 24;11(5):339. doi: 10.3390/jpm11050339 (PMC8145972; doi:10.3390/jpm11050339)

Journal of *Personalized Medicine*

MDPI

Supplementary Table S1. Kendall Correlations between Lipid Value and Percent of Total Calories

Group	Time	Total dhSM	Total SM	Total Cer	Total 2-hydroxy Cer	Total Hex-Cer	Total Lac-Cer
ECMO	Day 1	0.499	0.262	0.577	0.000	0.157	-0.052
ECMO	Day 3	0.158	0.293	0.000	0.586	0.293	-0.350
ECMO	Day 8	0.200	-0.200	0.467	-0.333	0.067	0.067
MODS	Day 1	N/A	N/A	N/A	N/A	N/A	N/A
MODS	Day 3	N/A	N/A	N/A	N/A	N/A	N/A
MODS	Day 8	N/A	-0.429	-0.214	-0.182	-0.214	-0.071

Notes: 2-hyroxy Cer: 2-hydroxy ceramide; Cer: ceramide; dhSM: dihydrosphingomyelin; Hex-Cer: hexosylceramides; Lac-Cer: lactosylceramides; SM: Sphingomyelin.

Supplementary Table S2: Correlations between lipid values and viral infection (yes/no) in patients over time; not adjusted for age or sex

Group	Time	Infection	Total dhSM	Total SM	Total Cer	Total hydroxy-Cer	Total Lac-Cer
MODS	Day 1	Bacterial	N/A	0.024	0.189	N/A	0.450
MODS	Day 3	Bacterial	N/A	-0.159	-0.106	0.447	0.080
MODS	Day 8	Bacterial	N/A	-0.436	-0.109	0.111	-0.109
MODS	Day 1	Viral	N/A	-0.382	-0.209	N/A	-0.247
MODS	Day 3	Viral	N/A	0.559	0.206	-0.309	0.118
MODS	Day 8	Viral	N/A	0.000	-0.109	0.056	0.000
ECMO	Day 1	Viral	0.361	0.049	-0.049	-0.447	-0.146
ECMO	Day 3	Viral	0.136	0.252	0.504	0.378	-0.065
ECMO	Day 8	Viral	-0.086	-0.086	-0.086	0.430	0.086

Notes: 2-hyroxy Cer: 2-hydroxy ceramide; Cer: ceramide; dhSM: dihydrosphingomyelin; Hex-Cer: hexosylceramides; Lac-Cer: lactosylceramides; SM: Sphingomyelin.

Supplementary Figure S1. Percent total cholesterol lipid species

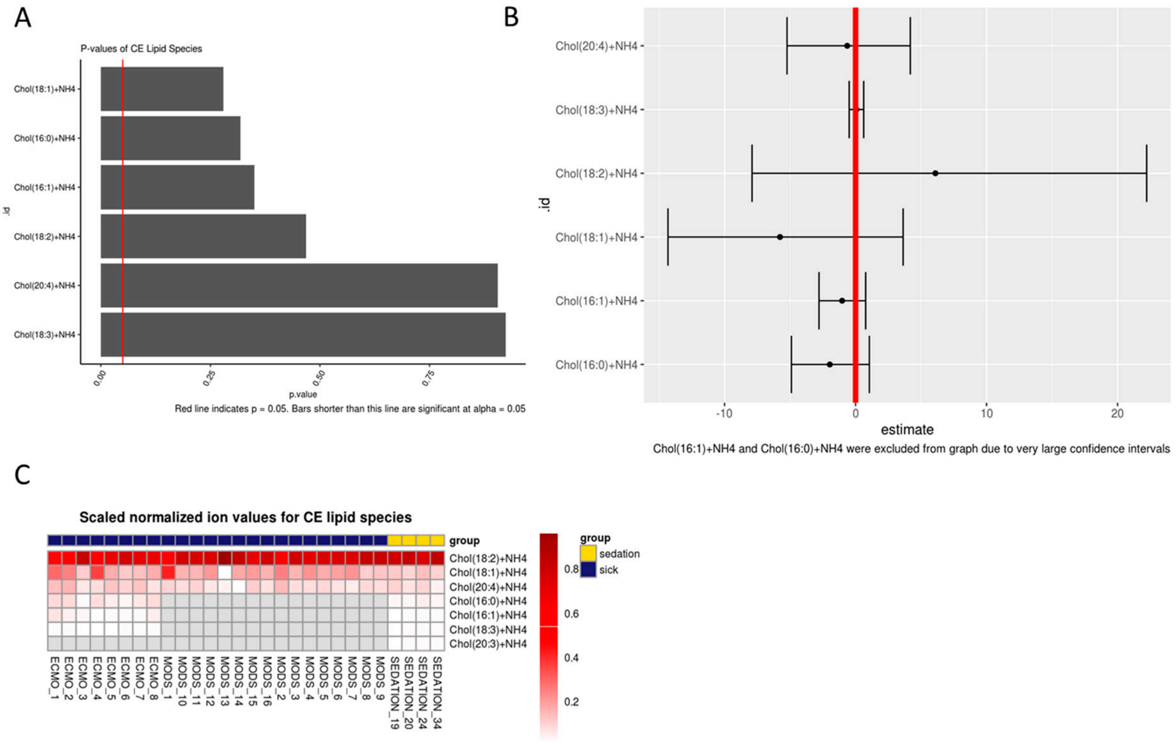

Supplementary Figure S2. Percent total triacylglycerol lipid species

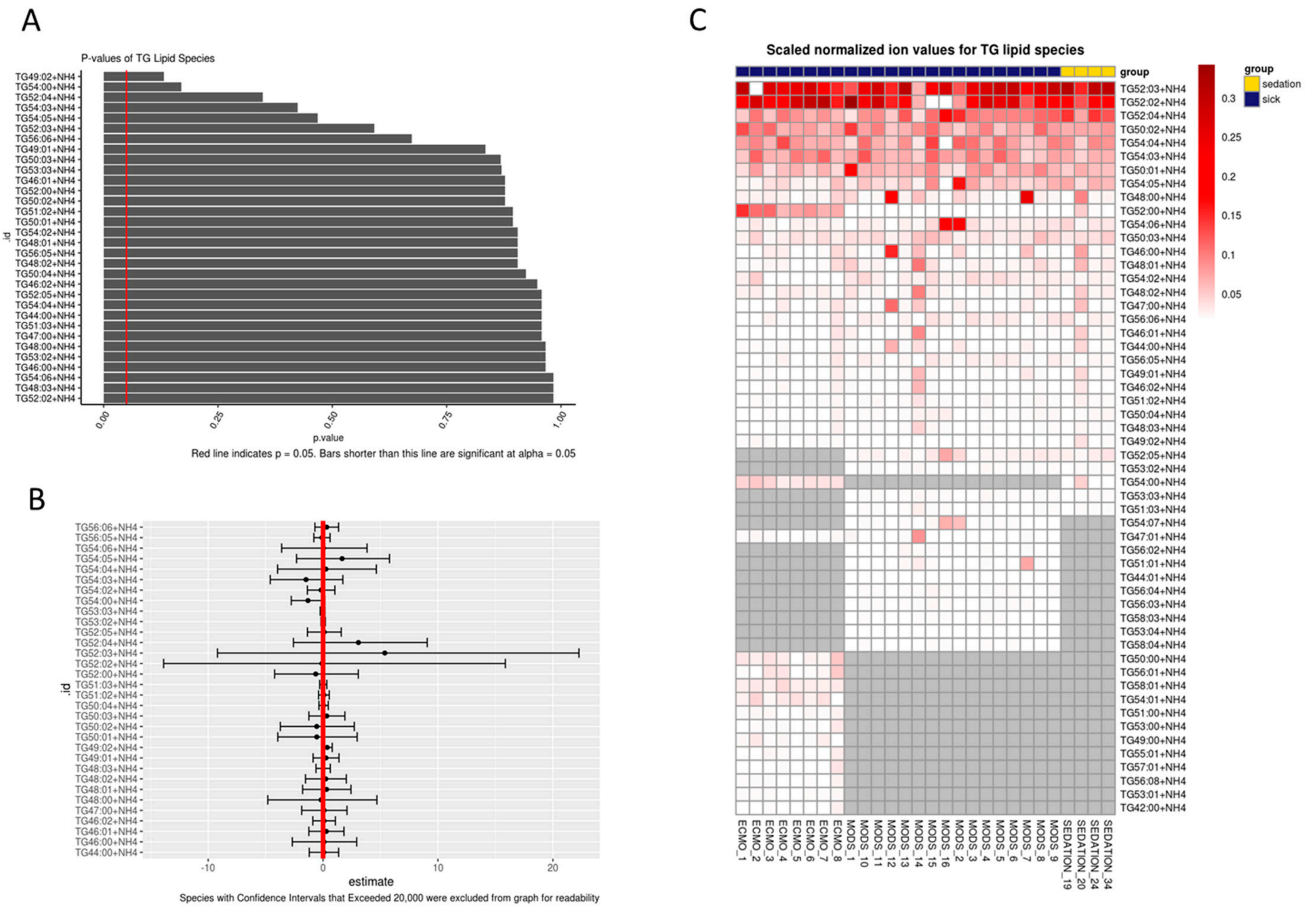

Supplementary Figure S3. Percent total diacylglycerol lipid species

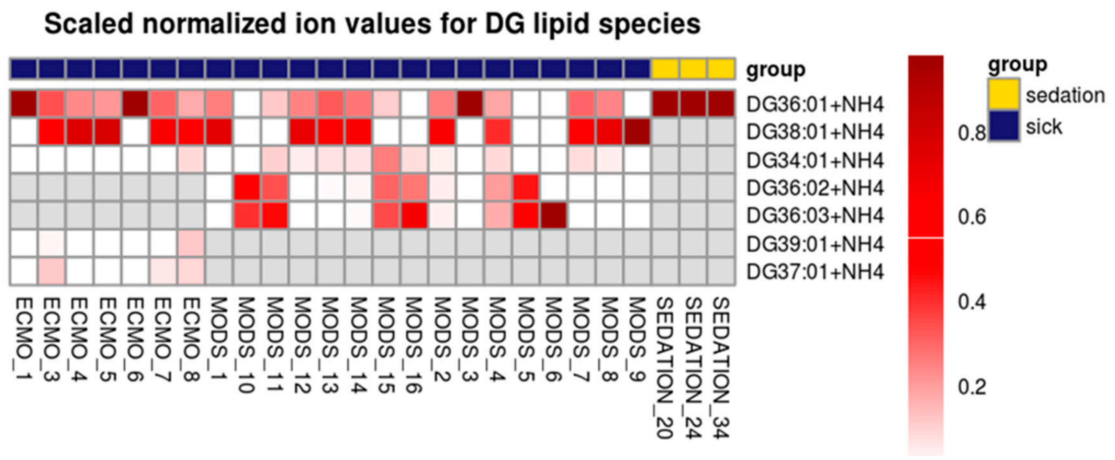

Supplement: Supplementary file 1 [file jpm-11-00339-s001.zip › jpm-1131836-supplementary.pdf]
